# High-density, high-frequency and large-scale electrohydrodynamic drop-on-demand jetting via a protruding polymer-based printhead design

**DOI:** 10.1038/s41378-024-00786-2

**Published:** 2024-11-05

**Authors:** Yongqing Duan, Weili Yang, Qiming Wang, Zhaoyang Sun, Haoyu Guo, Zhouping Yin

**Affiliations:** 1 State Key Laboratory of Intelligent Manufacturing Equipment and Technology, Huazhong University of Science and Technology, Wuhan, China; 2 Flexible Electronics Research Center, Huazhong University of Science and Technology, Wuhan, China

**Keywords:** Electrical and electronic engineering, Electronic devices

## Abstract

Electrohydrodynamic (EHD) printing has critical merits in micro/nanoscale additive manufacturing because of its ultrahigh resolution and wide ink compatibility, making it an advantageous choice for electronics manufacturing, high-resolution prototyping, and biological component fabrication. However, EHD printing is currently limited by its rather low throughput due to the lack of high-frequency and high-density multi-nozzle printheads. This paper presents a novel EHD printhead with a protruding polymer-based nozzle design. An insulated, hydrophobic, and protruding polymer nozzle array with an appropriate geometric structure can effectively address key problems in multi-nozzle jetting, such as electrical crosstalk, electrical discharge, liquid flooding, and nonuniform jetting. By investigating the influence of the electrical and geometric characteristics of the nozzle arrays on the electrical crosstalk behavior and fabricating the optimized nozzle array via MEMS technology, we achieve an EHD printhead with a large scale (256), high density (127 dpi), and high jetting frequency (23 kHz), and addressable jetting can be realized by adding independently controllable extractors underneath the nozzle array. Many functional materials, such as quantum dots, perovskite, and nanosilver inks, can be ejected into high-resolution patterns through the optimized nozzle array, demonstrating the great prospects of our designed printhead in electronics manufacturing. This MEMS-compatible printhead design lays the foundation for high-throughput fabrication of micro/nanostructures and promotes practical applications of EHD printing in functional electronics and biomedical/energy devices.

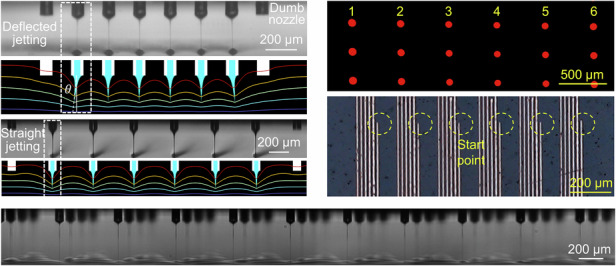

## Introduction

EHD printing utilizes high electrical forces to generate micro/nano jets or droplets and has recently attracted significant research interest^1^ owing to its critical advantages in printing resolution^2,3^ ( < 100 nm) and ink compatibility (applicable ink viscosity range of 1–10,000 cps). It is an advantageous choice for electronic fabrication^4^, high-resolution prototyping, and biological component fabrication^5^, and various functional inks, such as polymers^6^, proteins^7^, DNA^8,9^, quantum dots^10^, perovskites^11–14^, and silver inks^15,16^ have been deposited through the EHD technique into high-resolution 2D/3D structures^17–19^. However, current research on EHD printing uses a single nozzle to eject tiny droplets, and its low efficiency limits its application beyond the laboratory.

To realize high-throughput EHD printing, large nozzle arrays with high density and high jetting frequency are desperately needed. Increasing the nozzle array density aggravates electrical crosstalk^20^, resulting in jet deflection, inconsistent jetting frequency/droplet size, etc. To reduce crosstalk, the effects of the nozzle spacing, arrangement, and material have been studied experimentally and theoretically^20–23^. Adding dumb nozzles at the edge of the nozzle array and increasing the nozzle spacing are effective in decreasing the distortion of the electric field^24,25^. In addition, researchers have reported that dielectric nozzle materials are more conducive to reducing electrical crosstalk than metal nozzles^26^. For the jetting frequency, the capillary wave frequency of the meniscus is considered critical for the jetting frequency limit, which largely depends on the meniscus diameter^27^. Moreover, the flow rate, applied voltage, and ink viscosity also affect the jetting frequency^28^. In addition to density and frequency, addressable jetting of the nozzle array is critical for complex pattern deposition. Individually controllable syringe pumps^29^ or ring-shaped extractors^30,31^ have been used to achieve independent control of each nozzle. However, they are limited in response speed and integration density. Further investigations on the design of high-density, high-frequency, and addressable EHD printheads are still desperately needed.

The precise fabrication of EHD printheads with large-scale nozzles is crucial for practical application, which poses a challenge to the consistency of nozzles and flow channels. To increase the electric field strength and prevent the pendent drop from flooding, a needle-shaped nozzle array is preferred, but it poses difficulties for precise fabrication. Assembling several stainless steel nozzles or glass capillaries into a nozzle array is a simple and effective method, but these manually assembled nozzle arrays are limited in terms of the nozzle size, integration density, and nozzle consistency. To overcome these problems, high-precision silicon nozzle arrays fabricated via an etch-based process have gradually come into focus^32,33^. However, the conductivity of silicon makes the nozzle tip susceptible to electrical breakdown, which significantly reduces the nozzle lifetime. Instead, insulating materials such as glass and polymers are considered advantageous for avoiding the discharge phenomenon^34^, but they present new problems for high-precision fabrication. In addition to MEMS-based technology, special processing techniques such as laser etching^35^, micromachining^36^, and 3D printing^37,38^ have been explored in the fabrication of EHD nozzle arrays. Table S1 summarizes the nozzle arrays fabricated by different methods and their corresponding printing capabilities reported in the literature. Most multi-nozzle EHD printheads are currently designed for electrospray or electrospinning with limited capabilities. However, current multi-nozzle printheads are not well suited for drop-on-demand (DOD) EHD printing, which requires higher printing accuracy and a lower printing height. For example, silicon-based nozzles fabricated by established MEMS processes are prone to discharge at low printing heights owing to their inherent conductivity. The micro- and nanoprocessing technology for SiO_2_ materials is complex and expensive, and an additional hydrophobic layer is usually needed, resulting in low durability and print uniformity of the nozzle. At present, the design and manufacture of high-density, high-frequency and high-reliability printheads pose great challenges and urgency for the high-precision practical application of this technology.

In this work, we develop a high-density, high-frequency and large-scale EHD printhead by systematically optimizing the nozzle material, structure, and processing. The printhead is composed of a silicon/silica plate and a polymer nozzle tip. The insulating character of the nozzle array is advantageous for eliminating electrical discharge during printing, whereas the hydrophobic character of the polymer is helpful for preventing liquid flooding. The effects of the nozzle spacing, length, and print height on electrical crosstalk are systematically analyzed through experiments and simulations to reveal the crosstalk-free working diagram for printhead design. Large-scale and high-density nozzle arrays have been fabricated via photolithography technology. Their performance in terms of printing frequency, stability, addressability, and ink compatibility was then investigated. The applicability of our proposed printhead for high-precision EHD DOD printing technology was demonstrated, laying the foundation for its widespread application.

## Results and discussion

### Printhead design and manufacturing

**Fig. 1:**
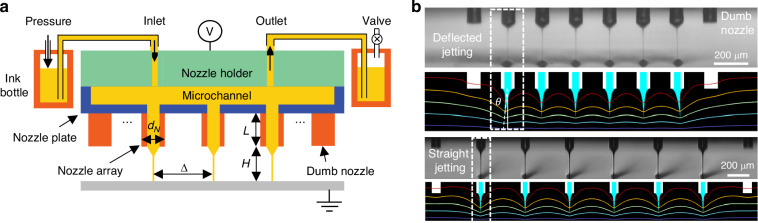
Arrayed EHD printhead and jetting process. **a** Schematic diagram of the EHD printhead; **b** simulation and experimental images of multi-nozzle jetting

The overall structure of the typical EHD printhead design is shown in Fig. 1a, which consists of a nozzle chip (a nozzle plate with a microchannel and protruding nozzle array), a nozzle holder (a glass plate to seal the microchannel), an inlet/outlet connector, ink bottles and a wire connected to high voltage. At the end of the nozzle array are dumb nozzles to suppress the electrical end effect. When a sufficiently high voltage is applied to the solution, the meniscuses at the nozzle tips deform into Taylor cones and start jetting as the electric force overcomes the surface tension. The key parameters for the printhead are the nozzle conductivity, diameter, length, spacing, and print height (*σ*_nozzle_, *d*_*N*_, *L*, Δ, and *H*, respectively), which can be tuned by varying the nozzle material, structure, and printing parameters. To clarify the influence of the key parameters on the concrete jetting behavior, *e.g*., cross-talk, integration density and jetting frequency, multi-nozzle jetting simulations are first performed. The multi-nozzle jetting simulations are conducted using a computational fluid dynamics model via COMSOL software (the details are shown in Figure S1 and Table S2). As shown in Fig. 1b, array jetting occurs with crosstalk (the jet from the edge nozzle is significantly deflected) and without crosstalk (all the jets straight downward without deflection) when Δ is 200 μm and 500 μm, respectively. The simulated jetting images are quite consistent with the experimental results under the same geometrical and material conditions, confirming the correctness of our presented model. This multi-nozzle jetting simulation provides important guidance for the latter printhead design.

**Fig. 2:**
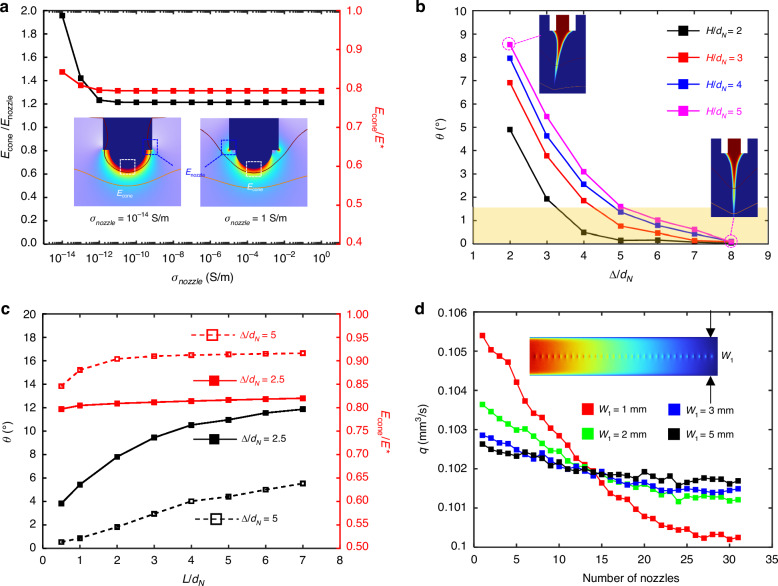
Design for nozzle array chip. **a** The ratio of *E*_cone_*/E*_nozzle_ (black line) and *E*_cone_/E* (red line) varying with the conductivity of the nozzle *σ*_nozzle_; **b** the deflection angle *θ* varying with the dimensionless nozzle spacing $\Delta/d_N$; **c** the influence of the nozzle length on the deflection angle *θ* and on the electric field intensity of the cone tip; **d** the influence of the channel width on the flow distribution of different nozzles

To clarify the influence of the nozzle conductivity on the spatial electric field distribution, we first analyzed the electric potential at the nozzle tip. The specific simulation process can be found in Supporting Information. Figure 2a shows that with increasing nozzle conductivity, both *E*_cone_/*E*_nozzle_ and *E*_cone_/E* gradually decrease; *E*_cone_, *E*_nozzle_ and E* correspond to the electric field at the meniscus cone tip, the nozzle tip, and the electric field intensity of a single nozzle under the same conditions, respectively. This decrease means that the insulating nozzle array has a larger electric force applied to the liquid meniscus to induce jetting, rather than a larger electric force concentrated at the nozzle edge that easily induces electrical breakdown. Therefore, nozzle arrays made of low-conductivity materials are preferred to reduce discharge and ensure stable printing.

Following the analysis of the material of the nozzle, further analysis was conducted on the multi-nozzle jetting behavior with different geometric structures, including the nozzle spacing, nozzle length, and print height. These factors directly affect the electric field distribution and the accuracy and uniformity of EHD printing. Since the edge nozzle has the largest jetting deviation (as shown in Fig. 1b), its deflection angle *θ* was used here to evaluate whether crosstalk exists. Figure 2b shows that *θ* decreases with the increase of Δ/*d*_*N*_ (nozzle spacing/nozzle diameter) and the decrease of *H*/*d*_N_ (print height/nozzle diameter), where the nozzle length is fixed at *L*/*d*_*N*_ = 1. Generally, *H*/*d*_N_ is within 2 ~ 10 for drop-on-demand printing; *H*/*d*_*N*_ values that are excessively small do not lead to a stable cone-jet because the Taylor cone is more easily pulled down onto the substrate; *H*/*d*_*N*_ values that are excessively large may cause serious jetting defection and produce satellite droplets. By taking *θ* < 1.5° as the limit for crosstalk-free printing, Δ/*d*_*N*_ ≥ 5 is preferred for uniform printing without crosstalk when *H*/*d*_*N*_ < 5.

In addition, the influence of the nozzle length is analyzed via simulation, as shown in Fig. 2c. A longer nozzle length corresponds to a more obvious deflection of the edge nozzle jet. To eliminate crosstalk, a shorter nozzle length is preferred. However, if the nozzle length is too small, the electric field strength at the nozzle tip will be reduced, making it difficult to realize EHD printing. After careful consideration, *L*/*d*_*N*_ < 2 is preferred. Considering all the above results, we find that Δ/*d*_*N*_ ≥ 5 and 1 < *L*/*d*_*N*_ < 2 are the optimal criteria for the multi-nozzle EHD printhead design. Within this range, the electrical crosstalk between nozzles is effectively reduced.

In addition to electrical crosstalk, the flow uniformity of different nozzles is also important for large-scale printing. Various microchannels, such as those with groove shapes (for single row nozzles, as shown in Fig. 2d) and branch shapes (for double row nozzles, as shown in Figure S2), are studied. For the groove shape type, the flow uniformity is affected by the cross-sectional area of the flow channel. The larger the channel cross-section is, the more uniform the flow rate of each nozzle. For a channel with a depth of 300 μm, as the width of the flow channel increases from 1 mm to 5 mm, the uniformity increases from 95% to 99%; this is because the increase in cross-sectional area reduces the effect of ink loss at the nozzles, thus making the flow more uniform across the nozzles. Similarly, for the branch-shaped channel, increasing the width of the main channel *W*_1_ has the same effect (as shown in Figure S2a). However, the width of the branch-shaped channel *W*_2_ has no significant effect on the flow uniformity because it hardly affects the pressure distribution along the direction of the nozzles (Figure S2b). Since the two types of channels have similar uniformities for the same main channel width, two channel shapes with a depth of 300 μm and a main channel width of 5 mm were finally adopted for single-row nozzles (groove shape) and double-row nozzles (branch shape) in this work. Because the area of a single chip cannot be too large, a groove-shaped channel is used for more rows of nozzles.

Considering the above simulation results, SU8 polymer is chosen as the nozzle material instead of silicon because it has rather low conductivity ( ~ 10^-13 ^S/m) to eliminate discharge, a high hydrophobic angle ( ~ 100° of water) to prevent unwanted flooding at the nozzle tip, good solvent resistance to ensure compatibility with different inks, and excellent compatibility with photolithography technology to ensure processing consistency. In general, the outer diameter of the nozzle is 80 μm, and the inner diameter is 40 μm.

**Fig. 3:**
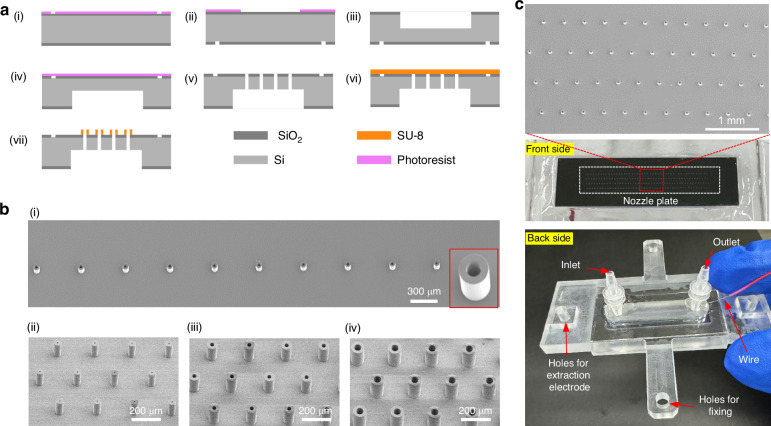
Preparation and integration of arrayed EHD printhead. **a** Fabrication process for EHD nozzle arrays; **b** SEM images of the multi-nozzle chip with different nozzle diameters and arrangements: (i) linear nozzle array with an 80-μm outer diameter and 40-μm inner diameter; (ii) multirow nozzle array with a 50-μm outer diameter and 10-μm inner diameter; (iii) multirow nozzle array with a 70-μm outer diameter and 30-μm inner diameter; (iv) multirow nozzle array with a 90-μm outer diameter and 50-μm inner diameter; **c** physical pictures of the printhead and its components. The top and bottom images are the front and back side views of the printhead, respectively

EHD printing chips with protruding polymer-based nozzles are fabricated via MEMS technology, and the specific fabrication process is shown in Fig. 3a and the Materials and methods section. In steps (i-v), the wafer was etched by photolithography and deep reactive ion etching to form alignment patterns (to facilitate subsequent precise alignment of the holes and nozzles), ink cavity microchannels, and microholes. In steps (vi-vii), the wafer was first cleaned by plasma etching and preheated to 80 °C to improve adhesion to the SU8 dry film. Then, a 100-μm-thick SU8 dry film was applied to the wafer and compacted with a dust-free roller. After prebaking, lithography, postbaking, development and hardening, cylindrical SU8 nozzles were fabricated. The fabricated nozzle plate is shown in Fig. 3b and Figure S4, and the nozzle arrangement, number, and inner/outer diameter can be easily tuned. The nozzle height is equal to the thickness of the polymer film, and this height can be tuned by using films with different thicknesses. After removing the individual printhead chip and bonding it to the nozzle holder and inlet/outlet connectors with UV-cured epoxy, an EHD printhead is formed, as shown in Fig. 3c.

### Analysis of the jetting performance

**Fig. 4:**
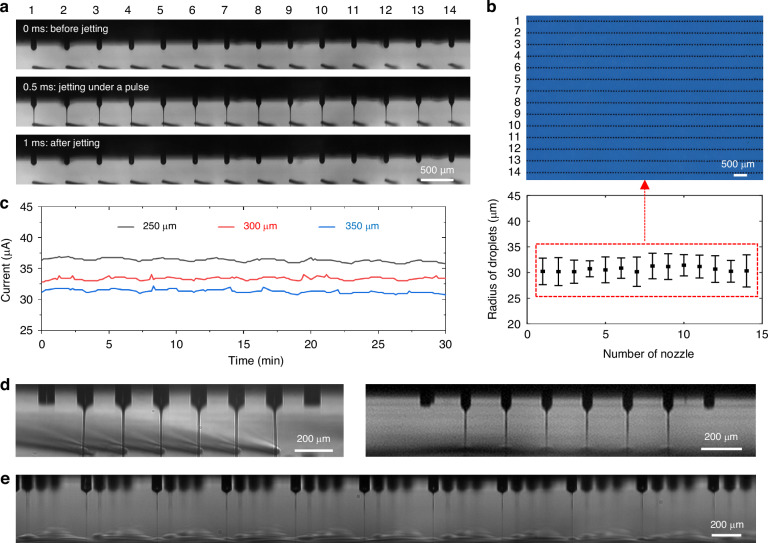
Stability and uniformity testing of arrayed EHD printhead. **a** Jetting images of a multi-nozzle array for one pulse; **b** corresponding printed droplets of the nozzle array presented in (**a**) and the diameter analysis of the printed droplets; **c** jetting current measured at different print heights over time; **d** jetting images of two nozzle arrays with the same nozzle spacing (200 μm) but different nozzle diameters (80 μm for the left and 45 μm for the right); **e** jetting image of 256 nozzles arranged in four rows

The jetting image of a nozzle array with a 500-μm nozzle spacing and an 80-μm nozzle diameter is shown in Fig. 4a, where all the jets are oriented vertically downward and there is no obvious deflection. In addition, the homogeneity and consistency of the jetting is high under the scrutiny of a high-speed camera. This finding is consistent with our presented simulation results that crosstalk-free printing can be achieved when Δ/*d*_*N*_ ≥ 5. By using the pulse voltage as the driving voltage, DOD printing can be achieved (Fig. 4b). We further analyzed the uniformity of the printed dots for each nozzle and found that a high uniformity of 98% was achieved between different nozzles. In addition, we also test the long-term printing stability, as shown in Fig. 4c. The measured current between the nozzle and substrate has tiny fluctuations, demonstrating that this printing process is very stable, which may benefit from the hydrophobic and insulating character of the SU8 nozzle to prevent discharge and flooding.

Conversely, Fig. 4d shows a jetting image of a nozzle array with 200 μm nozzle spacing and 80 μm diameter, where the jets in the center are oriented vertically downward, whereas the jet at the nozzle edge is slightly inclined ( < 5°). At this nozzle spacing, crosstalk cannot be completely suppressed, and the experimental results are consistent with the electric field simulation results in Fig. 2. Reducing the nozzle diameter is the most effective way to improve the nozzle density. By reducing the nozzle diameter from 80 μm to 45 μm, electrical crosstalk can be completely inhibited at the same nozzle spacing of 200 μm. Moreover, this nozzle design can be easily extended to a larger scale with different nozzle arrangements, as shown in Fig. 4e and Video S1, where 256 nozzles arranged in four rows can achieve stable jetting, and this number can easily be further expanded by MEMS technology to meet different manufacturing requirements.

**Fig. 5:**
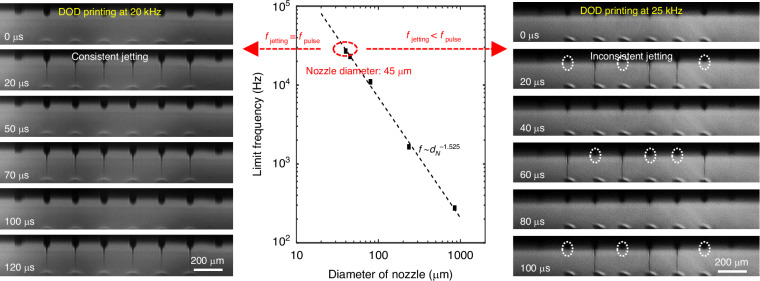
The variation in the maximum jetting frequency with the nozzle diameter. The left and right images are taken when the driving voltage frequency is below (left) and above (right) the limit frequency

In addition to increasing the nozzle integration density and number, enhancing the jetting frequency is also critical for high-throughput printing. For EHD jetting, the limit jetting frequency is considered to be dominated by the resonance frequency of the meniscus oscillation when the supplied flow rate is sufficient. It is highly related to the nozzle diameter and ink properties (*e.g*., surface tension and density)^39^. $\left(f=\sqrt{16\gamma /{\pi }^{2}{\rho d}_{N}^{3}}\right)$ Since ink properties vary widely for different solution applications, only the influence of the nozzle diameter is discussed here. For the nozzle array with an outer diameter of 45 μm, a high printing frequency limit of 23 kHz can be achieved with ethanol according to the above formula. Two specific examples and detailed frequency sweeping experiments can be seen in Fig. 5 and Video S2, where the jetting frequency can follow the pulse voltage frequency exactly within 23 kHz, and irregular jetting occurs when the driving frequency is above that limit (some cycles do not jet), and this result is quite consistent with the formula. To our knowledge, this frequency of over 20 kHz is the highest DOD EHD printing frequency reported in the literature. Similarly, a jetting frequency limit of ~10 kHz can be achieved when the outer diameter of the nozzle array is 80 μm (detailed frequency sweeping experiments can be seen in Video S3). We calculated the limit jetting frequency with respect to the nozzle diameter and found that the maximum jetting frequency follows *f* ~ *d*_*N*_^-1.525^, which is very close to *f* ~ *d*_*N*_^-1.5^. The error may be due to the meniscus diameter being slightly smaller than the nozzle diameter. Based on the formula, it is possible to achieve an ultrahigh jetting frequency of over 100 kHz by reducing the nozzle diameter to ~15 μm; this frequency is attractive even compared with commercial piezoelectric or thermal inkjet printing techniques. However, the ultrahigh jetting frequency corresponds to a smaller diameter, which makes high-precision manufacturing difficult and easily causes nozzle clogging; moreover, it places greater requirements on print height control and a high voltage/frequency power supply to ensure printing accuracy. Printheads with different limit jetting frequencies can be designed according to actual requirements.

**Fig. 6:**
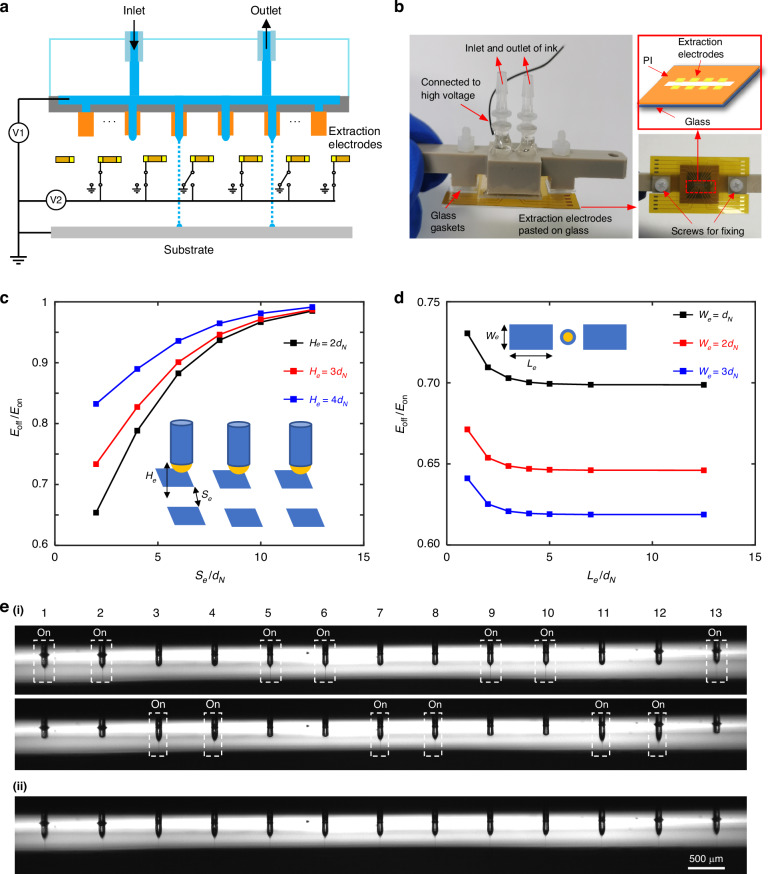
Using extraction electrodes to achieve addressable EHD printing. **a** Schematic of addressable EHD printhead; **b** physical image of addressable EHD printing; **c** effects of the spacing between the square electrode pairs *S*_*e*_ and the vertical distance between the nozzle tip and the electrode pairs *H*_*e*_ on the performance of independent control; **d** effects of the electrode length *L*_*e*_ and electrode width *W*_*e*_ on the performance of independent control; **e** jetting images of different nozzle combinations: (i) selective jetting of the nozzle array; (ii) all nozzles jetting simultaneously

The addressability of the nozzle array is critical for complex patterning, and extraction electrodes underneath each nozzle are applied here. By exerting a high voltage to the nozzle, a zero potential to the substrate, and a varying voltage to the extractor, the electric field strength of the meniscus is digitally controlled to realize addressability, as shown in Fig. 6a. The extraction electrodes are parallel copper electrodes encapsulated in PI. The extraction electrodes are first affixed to a glass substrate and then screwed to the nozzle array, as shown in Fig. 6b. To ensure independent control performance, we performed a detailed simulation design of the extraction electrodes. As shown in Figure S5, square electrodes are chosen to regulate the electric field in this work because they more easily cleaned of any spilled ink during use and are more tolerant of assembly positioning accuracy errors. Moreover, the effects of the electrode length *L*_*e*_ and electrode width *W*_*e*_ on the addressability performance are analyzed based on the design with Δ = 5*d*_*N*_. *E*_*off*_ and *E*_*on*_ are the electric field strengths at the tips of the turn-on and turn-off nozzles, respectively. The ratio represents the field strength ratio of the nozzles in different states. The larger the value is, the closer the field strengths of the nozzles in the turn-on and turn-off states, and the worse the addressability of the nozzle array. Figure 6c shows the effects of the spacing between the square electrode pairs *S*_*e*_ and the vertical distance between the nozzle tip and the electrode pairs *H*_*e*_ on the addressability performance. An increase in both *S*_*e*_ and *H*_*e*_ obviously reduces the control effect of the electrode pairs. However, in practical tests, excessively small *S*_*e*_ values can cause the jet to deflect toward the electrodes, resulting in nozzle failure. Excessively small *H*_*e*_ values can make assembly, observation, and cleaning difficult. However, Fig. 6d shows that it is more reasonable for *W*_*e*_ to be 2*d*_*N*_, which can ensure independent control performance and prevent the failure of two electrodes from being too close to each other. Since the length of the electrodes does not limit the design of other structures, *L*_*e*_ > 5*d*_*N*_ is sufficient. In combination with the test procedure and independent control performance, 2*d*_*N*_ < *S*_*e*_ < 3*d*_*N*_ and 2*d*_*N*_ < *H*_*e*_ < 3*d*_*N*_ are the appropriate electrode size ranges.

Triethylene glycol was selected as the printing solution. To ensure that all the nozzles can be fired smoothly, the voltage at the nozzle is set to 1600 V, which is slightly higher than the onset voltage ( ~1400 V). The extraction voltage should ensure that the electric field in the untriggered nozzle and the triggered nozzle is lower or higher than the critical value that induces nozzle jetting, respectively. When the extraction electrode is connected to 0 V or 550 V, the on/off state of each nozzle can be controlled independently, as shown in Fig. 6e, and this changing process is fast and repeatable, where the nozzle spacing Δ is 750 μm and the vertical distance from the nozzles to the extraction electrodes is 300 μm.

**Fig. 7:**
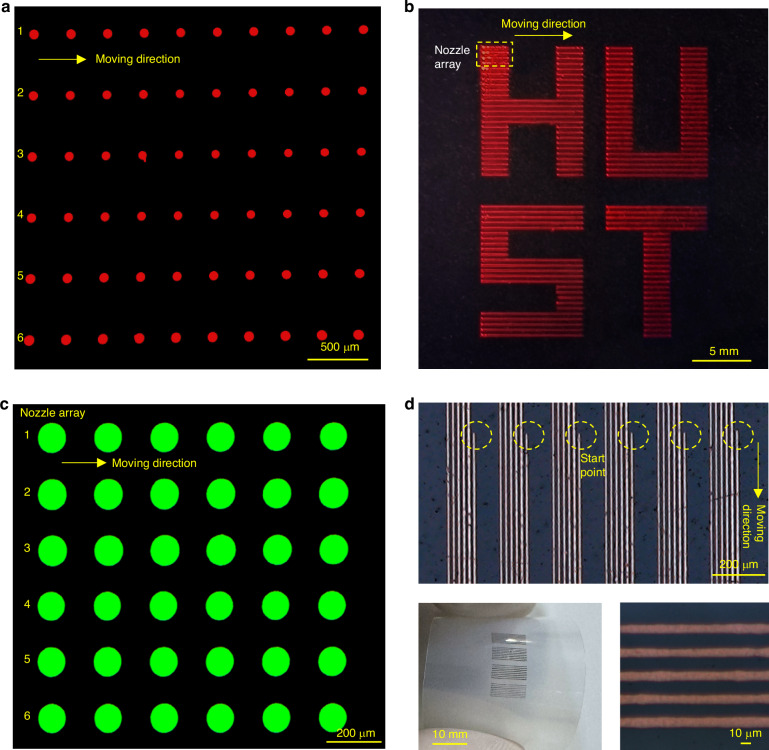
Printing test of various functional inks. **a** Luminescent lattice printed with a red quantum dot solution; **b** “HUST” printed with a red quantum dot solution; **c** luminescent lattice printed with a green perovskite solution; **d** printed lines with silver ink

We also test the ink compatibility of our proposed printhead. Several inks, such as ethanol, ethylene glycol, triethylene glycol, nanoparticle silver ink, and quantum dot solution, were tested, which demonstrated the good ink compatibility and printability of our designed nozzle array. Figure 7 shows some examples of the red quantum dot patterns, green perovskite patterns, and silver ink patterns printed via our multi-nozzle printhead. Figure 7a shows a quantum dot array printed via a 6-nozzle EHD printhead. By adjusting the amplitude, duty cycle, and frequency of the driving voltage, precise control of the droplet diameter can be achieved (Figure S6). By synchronizing the voltage switch with the movement of the platform, patterned printing can be achieved, as shown in Fig. 7b. For both perovskite and quantum dot solutions, the printed dot array can generate preset wavelengths of light under UV irradiation. By changing the solution formula, RGB patterns can be further printed. For silver ink printing, the ink has high conductivity, and it is easy to electrospray, so a small nozzle-to-collector distance is preferable to realize precise drop-on-demand printing. Silver lines with widths smaller than 10 μm demonstrate the high-resolution capability of our printhead. Moreover, the printhead is compatible with various substrates, such as rigid silicon, glass, and flexible polymer (Fig. 7d). They exhibit good printing resolution, accuracy and uniformity and have great potential in printed electronics applications.

The above test results prove that the EHD printhead can be designed on a large scale with high density and frequency; moreover, it is compatible with a wide range of solutions, and it is capable of generating droplets with high uniformity and high resolution. Additionally, this nozzle array can be easily extended to a larger scale and smaller nozzle diameter, which greatly supports the industrial application of EHD printing.

## Conclusion

In this study, the design and validation of a high-throughput EHD printhead is proposed. The use of insulating and hydrophobic polymers as protruding nozzle materials can greatly reduce unwanted discharge and liquid flooding. The influences of the nozzle diameter, spacing, length, and print height on the deflection of the jet are comprehensively investigated through simulations and experiments, and a crosstalk-free regime for printhead design is presented. The nozzle array fabricated via the photolithography-based technique can realize high-frequency (23 kHz), high-density (127 dpi for a single row), and large-scale (256) printing. To the best of our knowledge, this frequency and nozzle number are the highest for DOD EHD printing reported in the literature; moreover, these characteristics can be easily extended by decreasing the nozzle diameter and integrating more nozzles. It is also compatible with various inks, such as quantum dots, perovskite and silver ink, and can achieve addressable printing by adding extractors underneath the nozzle array. This nozzle design provides significant guidance for EHD-based high-precision micro/nanomanufacturing, and the high-throughput printhead shows great potential in electronics manufacturing and biomedical applications.

## Materials and methods

### Preparation of the inks

Ethanol, ethylene glycol, triethylene glycol, and nanoparticle silver ink (Silverjet DGP-40LT-15C) were purchased from Sigma Co., Ltd. All reagents were used without further purification. The methods for the preparation of the red quantum dot solution and perovskite solution are described in ref. ^40^.

### Fabrication of the EHD printhead

A high-resistivity silicon wafer with a 2-μm silica layer and a thickness of 500 μm was selected as the chip substrate, considering the advantages of a high-resistivity silica layer in preventing electrical discharge. First, the wafer is cleaned according to the RCA standard to ensure its surface smoothness and cleanness. In step (i), the wafer is etched by photolithography (photoresist EPI680 with a spin coating thickness of 2.7 μm) and deep reactive ion etching to form an alignment pattern (to facilitate subsequent precise alignment of holes and nozzles). In steps (ii-iii), the wafer is etched by photolithography (photoresist AZ 4620 with a spin coating thickness of 9 μm for microchannels ad 6 μm for microholes) and deep reactive ion etching to form microchannels and microholes. In steps (iv-vii), the surface of the wafer is cleaned by plasma etching, and the wafer is preheated to 80 °C (to improve adhesion to the SU8 dry film). Then, a 100-μm thick SU8 dry film is applied to the wafer and compacted with a dust-free roller. After prebaking (85 °C, 5 min), lithography (exposure at 11 mW/cm^2^ for 80 s), postbaking (95 °C, 10 min), development and hardening (150 °C, 60 min), a cylindrical SU8 nozzle was fabricated. Here, dry film was chosen because wet film photoresists are prone to clogging the holes during spin-coating and photolithography. To ensure that no debris is generated and that the surface finish of the wafer is not damaged when the wafer is split, the wafer is cut into small pieces via laser stealth cutting instead of mechanical cutting. After the individual printhead chip is removed and bonded to the nozzle holder and inlet/outlet connectors with special UV-cured epoxy, an EHD printhead is formed.

### Jetting test of the proposed EHD printhead

Figure S3 shows a photograph of the experimental platform. The ink supply system includes a pressure controller and several ink bottles. The pressure controller (OB1 MK3 + , ELVEFLOW, France) provides a stable air pressure supply to the ink bottle, which in turn is capable of pumping ink into the printhead in a stable manner. The voltage waveform is generated by a signal generator and output to a high-voltage amplifier (AMT-10B10, Matsusada, Japan), which is then connected to the nozzle via a wire. A DC voltage source (DW-P403, Dongwen, China) can be connected to the extraction electrodes for addressable EHD printing. The high-speed camera (i-speed 508, IX Cameras, UK) and light source form the observation system. The assembled nozzle can be coupled to the printhead fixture with screws, and the print height and the angle between the nozzle chip and the substrate are adjusted (to make the distance between each nozzle and the substrate as equal as possible, ensuring the uniformity of the electric field). The movement of the moving platform in conjunction with the voltage signal enables the printing of patterns.

Before printing, nozzle leveling, and ink injection should be completed first. During nozzle leveling, the nozzle surface should be highly parallel to the collector, as this directly affects the uniformity of the spatial electric field distribution. A difference in the nozzle-to-collector distance, such as 10 μm, can induce asynchronous injection between different nozzles. For the ink injection process, stable pressure is output by the pressure controller, and the ink in the ink bottle is pumped into the printhead. Then, the valve is closed when the air in the printhead is exhausted (it should be ensured that there are no bubbles left in the printhead, as tiny bubbles can easily cause nozzle blockage and print failure). Afterward, the pressure should be carefully adjusted to ensure that all nozzles have a stable meniscus at the nozzle tip (in this study, ethanol with a viscosity of 1 cP requires a pressure of approximately 20 mbar, whereas triethylene glycol with a viscosity of 40 cP requires a pressure of approximately 35 mbar), and the high voltage should be within an appropriate range to maintain Taylor cone formation and micro/nano jet printing. Nozzle lifetime is also an important characteristic. In the printing process, nozzle maintenance is very important. After printing experiments, we usually use deionized water to clean the nozzle and a nitrogen flow to force out all the gas inside the nozzle. We also keep it in a dust-free environment. Owing to the good chemical stability of the SU8 material, the lifespan of the nozzle can reach several months. However, as the small cylindrical nozzle is fragile, external forces can easily damage the nozzle structure, and dust in the environment can block the nozzle. Therefore, conducting experiments in a clean room and carefully cleaning after use will help improve the nozzle’s service life.

## Supplementary information

Supplementary Information

Video S1

Video S1

Video S1
