# Supplementary material for: High-density, high-frequency and large-scale electrohydrodynamic drop-on-demand jetting via a protruding polymer-based printhead design
Source: Microsyst Nanoeng. 2024 Nov 5;10:163. doi: 10.1038/s41378-024-00786-2 (PMC11535313; doi:10.1038/s41378-024-00786-2)
Supplement: Supplementary file 1 — Supplementary Information [file 41378_2024_786_MOESM1_ESM.docx]

Supplementary Information

High-density, high-frequency and large-scale electrohydrodynamic drop-on-demand jetting via protruding polymer-based printhead design

Yongqing Duan^1,2^, Weili Yang^1^, Qiming Wang^1^, Zhaoyang Sun^1^, Haoyu Guo^1^ and Zhouping Yin^1,2^

Correspondence: Yongqing Duan (duanyongqing@hust.edu.cn)

^1^ State Key Laboratory of Intelligent Manufacturing Equipment and Technology, Huazhong University of Science and Technology, Wuhan 430074, China

^2^ Flexible Electronics Research Center, Huazhong University of Science and Technology, Wuhan 430074, China

**This file includes:**

Figures S1-S6.

Tables S1-S2.

Videos S1-S3.

Details of numerical simulation.

Reference.

**Figures**


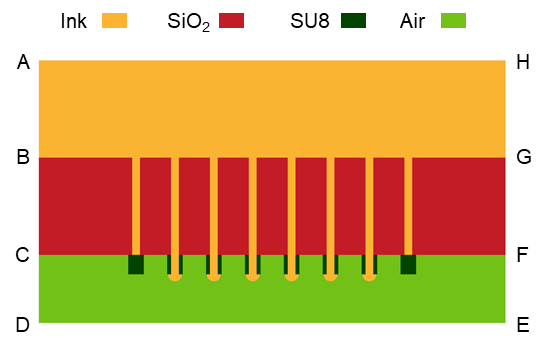


**Figure S1. The schematic diagram of the calculation domain.**

**
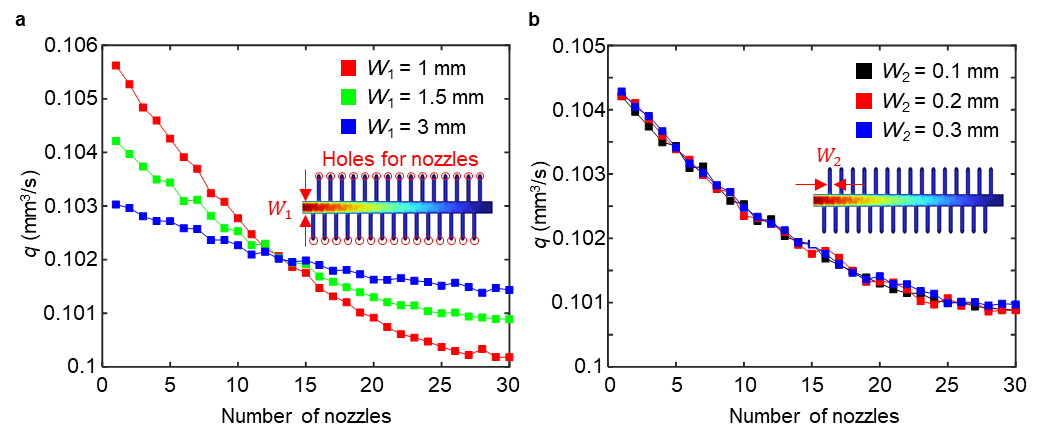
**

**Figure S2. For printhead with branch shape channel,** **the effect of the width of channels on the distribution of flow: (a)** Width of the main flow channel *W*_1_**; (b)** Width of branch channels *W*_2_**.**


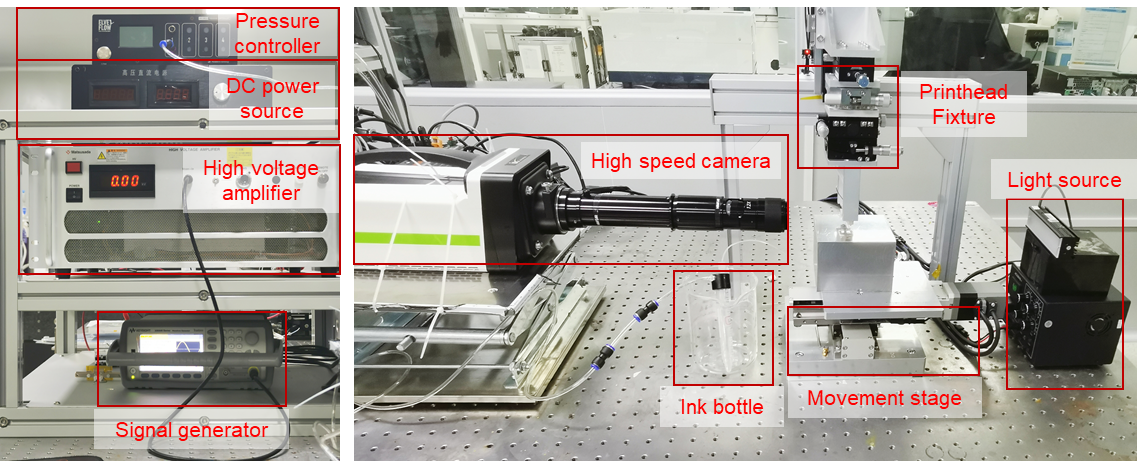


**Figure S3. The photo of the experimental platform.**

**
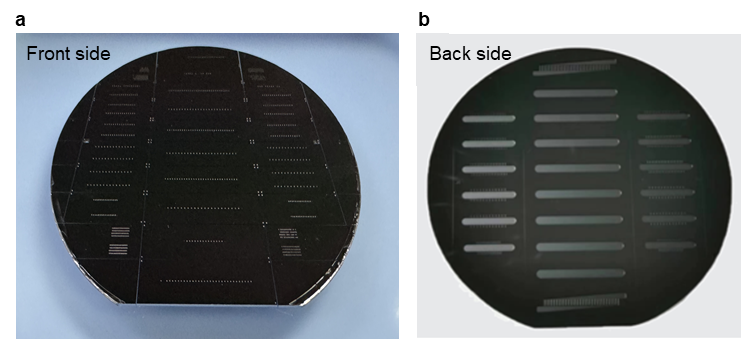
**

**Figure S4. The photos of the whole wafer for the printhead chips: (a)** the front side; **(b)** the back side.

**
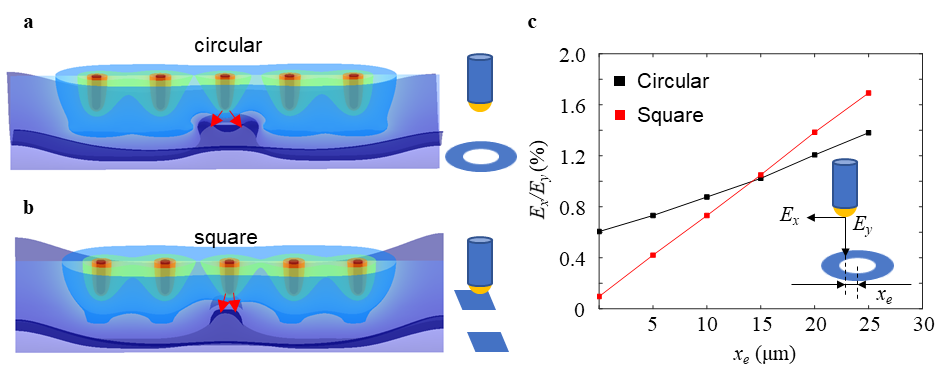
**

**Figure S5. Electric field distribution with different electrode shapes: (a)** Circular**; (b)** Square**; (c)** The effect of *x_e_* on *E_x_*/*E_y_*.

To ensure the independent control performance, the extraction electrodes were designed by simulation, and the shape, size, and position of the extraction electrodes are discussed here. First, the selection of the extraction electrode geometry is discussed based on the alignment error *x_e_* (the alignment error between the nozzle and the electrode center). Figure S5a and Figure S5b show the electric field when only the center nozzle is turned on for the two electrode shapes, respectively. And Figure S5c shows the changes in *E_x_*/*E_y_* (*E_x_* and *E_y_* are the transverse and vertical electric fields at the nozzle tip, respectively) as *x_e_* increases. It can be observed that *E_x_* of the square electrode is smaller than that of the circular electrode when *x_e_* < 15 μm. In the experiment, the typical *x_e_* is less than 10 μm. Therefore, the *E_x_* generated by the square extraction electrode is smaller. In addition, circular electrodes are difficult to apply to high-density nozzle arrays due to geometric constraints along the direction of nozzle orientation. Therefore, in this paper, square electrodes are chosen to regulate the electric field because of their advantages for printhead cleanliness and improving nozzle density.

**
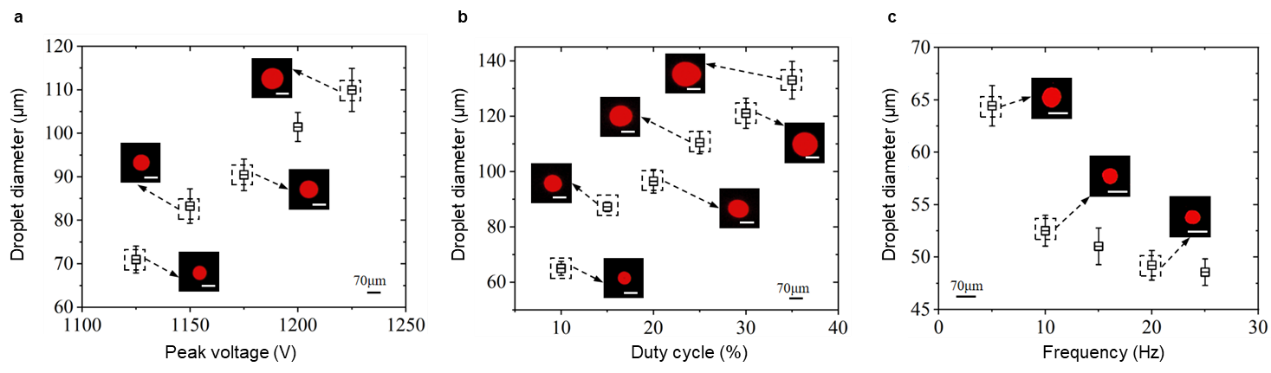
**

**Figure S6. The influence of driving voltage on the droplet diameter. (a)** Peak voltage; **(b)** Duty cycle; **(c)** Frequency.

**Table**

**Table S1.** The multi-nozzle electrohydrodynamic printhead in literature.

| Printing mode | Manufacturing process | Nozzle  material | Nozzle  size | Nozzle spacing | Number of nozzles | Jetting frequency (Hz) | Ref |
| --- | --- | --- | --- | --- | --- | --- | --- |
| Electrospray | MEMS | Si | ID: 20 μm | 250 μm | / | / | 1 |
|  | MEMS | Si | OD: 210 μm  ID: 60 μm | 675 μm | 19 | / | 2 |
|  | CNC | Metal, polymer | OD: 120 μm  ID: 50 μm | 500 μm | 51 | / | 3 |
|  | Laser-based micromachining | Si | OD: 300 μm  ID: 55 μm | 750 μm | 16 | / | 4 |
|  | Manual assembly | Steel | OD: 1.5 mm  ID: 0.5 mm | >1 mm | 3 | / | 5 |
|  | UV-embossing | Resin | OD: 880 μm  ID: 60 μm | 4 mm | 5 | / | 6 |
| Electrospinning | 3D printing | Resin | >1 mm | >1 mm | 17 | / | 7 |
| Drop-on-demand EHD printing | MEMS | Si | OD: 120 μm  ID: 50 μm | >1 mm | 3 | 20 | 8 |
|  | Glass reflow | SiO_2_ | OD: 100 μm  ID: 20 μm | 1 mm | 16 | 10 k | 9 |
|  | Sand blasting | SiO_2_ | OD: 100 μm  ID: 60 μm | 3 mm | 4 | 10 k | 10 |
|  | Manual | SiO_2_ | OD: 2 μm | >1 mm | 4 | 67 | 11 |
|  | MEMS | SU8 | OD: 80 μm  ID: 40 μm/  OD: 45 μm  ID: 15 μm | 400 μm/  200 μm | 256 | 23 k | This work |

**Table S2. Boundary conditions.**

| Boundary | Type | Electric boundary | Hydrodynamic boundary |
| --- | --- | --- | --- |
| AH | Inlet | $U=U_{0}$ | $u=0$  $v=v_{0}$ |
| DE | Ground(wall) | $U=0$ | $\boldsymbol{v}=0$ |
| CD, EF | Outlet | / | $p=0$ |

**Videos**

1. Video S1: the printing height is 340 μm, the rectangular pulsed voltage is 1350 ± 200 V, the jetting frequency is 100 Hz, and the duty cycle is 5%. The spacing of nozzles is 500 μm. The solution used in the video is ethanol. The video was shot at 5000 fps.

1. Video S2: the printing height is 200 μm. The spacing of nozzles is 200 μm. The solution used in the video is ethanol. The video was shot at 100000 fps. The video playback rate is 10 fps.

1. Video S3: the printing height is 300 μm. The spacing of nozzles is 400 μm. The solution used in the video is ethanol. The video was shot at 40000 fps. The video playback rate is 10 fps.

**Details of numerical simulation**

The motion of the fluid in the electric field is governed by the Navier–Stokes equations:

|  | $\nabla\cdot\mathbf{v}=0$ | (1) |
| --- | --- | --- |
|  | $\rho\frac{\partial\mathbf{v}}{\partial t}+\text{ρ}\text{v}\text{∙∇}\text{v}\text{=-∇}\text{p}\text{+}\mu\nabla^{2}\mathbf{v+}\mathbf{f}_{\mathbf{e}}\boldsymbol{+}\mathbf{f}_{\mathbf{st}}$ | (2) |

where $\rho$, $\mathbf{v}$, $p$, $\mu$ and $\gamma$ are the density, velocity, pressure, viscosity, and surface tension coefficient of the fluids, respectively. $\mathbf{f}_{\mathbf{e}}$ is the electric volume force acting on the fluid. $\mathbf{f}_{\mathbf{st}}$ is the surface tension.

The Level-Set (LS) method is used to track the interface of the two-phase flow. The LS method uses a signed distance function $\phi$ to capture the interface, where $\phi$ is 0 in air and 1 in ink. The following equation describes the convection of the reinitialized level set function:

|  | $\frac{\partial\phi}{\partial t}+\mathbf{v}\cdot\nabla\phi+\gamma_{ls}\left[ \nabla\cdot\left( \phi\left( 1-\phi\right)\frac{\nabla\phi}{\left\vert\nabla\phi\right\vert} \right)-\varepsilon_{ls}\text{∇∙∇}\phi\right]=0$ | (3) |
| --- | --- | --- |

where $\varepsilon_{ls}$ is the interface thickness control parameter, which is proportional to the thickness of the transition layer. In our model, $\varepsilon_{ls}= h_{c}/2$, where $h_{c}$ is the typical mesh size of the droplet at the tip of the nozzles. The parameter $\gamma_{ls}$ determines the amount of reinitialization^12,13^.

The surface tension force is computed as:

|  | $\mathbf{f}_{\mathbf{st}}=\gamma\delta\kappa\mathbf{n}$ | (4) |
| --- | --- | --- |

where $\mathbf{n}\mathbf{=}{\text{∇}\phi}/\left| \nabla\phi\right|$ is the interface normal, $\gamma$ is the surface tension coefficient, $\kappa=-\text{∇∙}\mathbf{n}$ is the curvature, and $\delta$ is equal to a Dirac delta function that is nonzero only at the fluid interface. The delta function is approximated by:

|  | $\delta=6\left\vert\phi\left( 1-\phi\right) \right\vert\left\vert\nabla\phi\right\vert$ | (5) |
| --- | --- | --- |

And the jumps in density $\rho$, viscosity $\mu$, dielectric constant $\varepsilon$ and conductivity $\sigma$ across the interface are smoothed by the definitions:

|  | $\rho=\rho_{air}+(\rho_{ink}-\rho_{air})\phi$ | (6) |
| --- | --- | --- |
|  | $\mu=\mu_{air}+(\mu_{ink}-\mu_{air})\phi$ | (7) |
|  | $\varepsilon=\varepsilon_{air}+(\varepsilon_{ink}-\varepsilon_{air})\phi$ | (8) |
|  | $\sigma=\sigma_{air}+(\sigma_{ink}-\sigma_{air})\phi$ | (9) |

In the LS method, $\mathbf{f}_{\mathbf{e}}$ can be derived from the electrostatic Maxwell stress tensor:

|  | $\mathbf{f}_{\mathbf{e}}=q\text{E}-\frac{\text{1}}{\text{2}}\left\vert\mathbf{E} \right\vert^{2}\nabla\varepsilon$ | (10) |
| --- | --- | --- |

The electric field is related to the conductivity $\sigma$ and free charge density $q$ by the charge conservation equation:

|  | $\frac{\partial q}{\partial t}+\nabla\cdot\left( \sigma\mathbf{E}+q\text{v} \right)=0$ | (11) |
| --- | --- | --- |

where $q=\nabla\text{∙}(\varepsilon\text{E})$. Since the charge relaxation time scale in this paper is assumed to be very small (~2×10^-6^ s) and charges are considered to be static, Eq. (11) becomes:

|  | $\nabla\cdot\left( \sigma\mathbf{E} \right)=0$ | (12) |
| --- | --- | --- |

where $\text{∇×}\mathbf{E}=0$.

**Reference**

1 Krpoun, R. & Shea, H. R. A method to determine the onset voltage of single and arrays of electrospray emitters. *J Appl Phys* **104**, 064511, (2008).

2 Almería, B., Deng, W., Fahmy, T. M. & Gomez, A. Controlling the morphology of electrospray-generated PLGA microparticles for drug delivery. *J Colloid Interf Sci* **343**, 125-133, (2010).

3 Lojewski, B., Yang, W., Duan, H., Xu, C. & Deng, W. Design, Fabrication, and Characterization of Linear Multiplexed Electrospray Atomizers Micro-Machined from Metal and Polymers. *Aerosol Sci Tech* **47**, 146-152, (2013).

4 Li, L., Yang, W., Zhao, X. & Deng, W. Multiplexed electrospray emitters fabricated by rapid laser micromachining. *J Aerosol Sci* **150**, (2020).

5 Quang Tran Si, B., Byun, D. & Lee, S. Experimental and theoretical study of a cone-jet for an electrospray microthruster considering the interference effect in an array of nozzles. *J Aerosol Sci* **38**, 924-934, (2007).

6 Jeong, J. H. *et al.* Multiplexed electrospraying of water in cone-jet mode using a UV-embossed pyramidal micronozzle film. *Microsystems & Nanoengineering* **8**, 110, (2022).

7 Garcia-Lopez, E., Olvera-Trejo, D. & Velasquez-Garcia, L. F. 3D printed multiplexed electrospinning sources for large-scale production of aligned nanofiber mats with small diameter spread. *Nanotechnology* **28**, 425302, (2017).

8 Pan, Y., Chen, X., Zeng, L., Huang, Y. & Yin, Z. Fabrication and evaluation of a protruding Si-based printhead for electrohydrodynamic jet printing. *J Micromech Microeng* **27**, 125004, (2017).

9 Lee, K. I. *et al.* in *2014 IEEE 27th International Conference on Micro Electro Mechanical Systems (MEMS).* 963-966 (IEEE).

10 Lee, K. *et al.* in *2013 IEEE 26th International Conference on Micro Electro Mechanical Systems (MEMS).* 1165-1168 (IEEE).

11 Takagi, M. Electrohydrodynamic jet printing: advancements in manufacturing applications. (2013).

12 Olsson, E. & Kreiss, G. A conservative level set method for two phase flow. *J Comput Phys* **210**, 225-246, (2005).

13 Olsson, E., Kreiss, G. & Zahedi, S. A conservative level set method for two phase flow II. *J Comput Phys* **225**, 785-807, (2007).
